# Development and Validation of a Multimodal Wearable Belt for Abdominal Biosignal Monitoring with Application to Irritable Bowel Syndrome

**DOI:** 10.3390/mi16111255

**Published:** 2025-11-01

**Authors:** Amir Mohammad Karimi Forood, Sibi M. Pandian, Riley Q. McNaboe, Thuany De Carvalho Lachos, Daniel Octavio Lantigua, Hugo F. Posada-Quintero

**Affiliations:** Department of Biomedical Engineering, University of Connecticut, Storrs, CT 06269, USA; karimi@uconn.edu (A.M.K.F.); sibi.pandian@uconn.edu (S.M.P.);

**Keywords:** irritable bowel syndrome, wearable biosignal acquisition, electrodermal activity, electromyography, electrocardiogram, signal processing

## Abstract

Visceral pain in Irritable Bowel Syndrome (IBS) is difficult to evaluate objectively due to its complex physiological nature and lack of reliable biomarkers. Given the complexity of IBS, a multimodal physiological monitoring approach, combining electrodermal activity (EDA), electrocardiogram (ECG), and surface electromyography (sEMG), offers a promising approach to capture the autonomic and muscular responses linked to visceral pain. However, no existing wearable device supports simultaneous EDA, ECG, and sEMG acquisition from the abdomen in a format suitable for long-term, real-world use. This study presents the development and validation of a novel wearable belt for concurrent ECG, sEMG, and EDA monitoring, with EDA measured at both the torso and wrist. The system was built using modified BITalino platforms with custom-fabricated reusable electrodes and Bluetooth connectivity for real-time smartphone display. Signal quality was validated against laboratory-grade systems in 20 healthy participants during a four-stage protocol involving cognitive, orthostatic, muscular, and combined stress tasks. Time and frequency-domain analyses showed high correlations and comparable spectral features across all modalities. The belt maintained stable skin contact even during movement-intensive tasks. By enabling anatomically targeted, multimodal data acquisition, this wearable system supports real-world visceral pain assessment in IBS and is ready for deployment in ambulatory and home-based monitoring scenarios.

## 1. Introduction

Irritable bowel syndrome (IBS) is a common functional gastrointestinal disorder characterized by chronic abdominal pain and altered bowel habits. Because diagnosis relies on symptom-based criteria rather than objective biomarkers, assessing physiological correlates of visceral pain and autonomic imbalance remains a major challenge. Dysfunction of the autonomic nervous system (ANS) has been associated with IBS-related visceral pain [1]. The ANS is the primary pathway in brain-gut communication, manifesting emotional and psychological states in the body. This makes it particularly relevant to IBS, as it is associated with malfunctions in the brain-gut neural axis and has a strong emotional component. The ANS comprises the sympathetic (SNS) and parasympathetic (PNS) nervous systems, and IBS is reportedly associated with an unchecked SNS predominance and desensitized PNS activity [2]. Therefore, the SNS and PNS are promising targets for developing sensitive and robust IBS pain biomarkers. We have demonstrated the non-invasive quantification of both SNS and PNS activities with time- and frequency-domain analysis of EDA [3,4] and ECG [5,6]. In addition, our studies and others’ indicate that the EMG of abdominal muscles can be a metric of visceral pain to differentiate IBS sufferers from control populations [7,8,9].

Since many health conditions involve interactions among multiple physiological systems, relying on a single biosignal often provides an incomplete picture. This has led to growing interest in multimodal biosignal acquisition, where signals are recorded in parallel to improve diagnostic accuracy and physiological insight [10]. IBS exemplifies the need for such an approach, as it lacks definitive biomarkers and requires reliance on Rome IV symptom-based criteria and exclusion of organic disease [11]. Integrating EDA, ECG, and sEMG will enable a comprehensive understanding of IBS symptom mechanisms and supports the development of personalized, data-driven treatment strategies.

Multimodal biosignal monitoring offers a more objective method for evaluating physiological disruptions associated with IBS. EDA reflects sympathetic nervous system activity and is commonly employed to assess emotional arousal and stress reactivity [12]. ECG, particularly through heart rate variability (HRV) analysis, captures the balance between sympathetic and parasympathetic branches of the autonomic nervous system [13]. sEMG measures muscle activity and is frequently used to evaluate motor function and tension in abdominal and skeletal muscles [7]. This direction is further reinforced by advances in wearable bioelectronics that allow real-time, organ-specific monitoring and potentially closed-loop interventions [14].

Current devices are largely restricted to lab settings, involve bulky equipment, or require multiple separate devices. Furthermore, reliance on adhesive gel electrodes limits long-term wear due to discomfort, skin irritation, and signal degradation, reducing feasibility for daily monitoring in real-world contexts [15,16]. Recent progress in low-power bioelectronics and energy-autonomous systems has accelerated the advancement of multimodal healthcare platforms capable of continuously monitoring physiological signals in real-world settings [17,18]. These developments emphasize flexible, lightweight, and sustainable designs that enhance user comfort while maintaining high signal fidelity, reflecting a broader shift toward integrating multiple sensing modalities within compact wearable systems [10,19,20]. In alignment with these emerging trends, we developed a compact wearable abdominal belt system that integrates multimodal biosensing capabilities, including one ECG channel, two EMG channels, and two EDA channels, as well as an embedded accelerometer. In addition, a single EDA channel is implemented on the wristband to complement the belt-based measurements.

As the first multimodal system developed and validated for IBS-related physiological monitoring, correlation-based analyses were selected as suitable first-level validation metrics to establish feasibility and physiological consistency, providing a foundational framework for future multimodal studies.

## 2. Materials and Methods

The wearable abdominal belt system is based on modified BITalino boards optimized for our sensor layout and mechanical constraints [21]. Wireless data transmission to smartphones enables real-time signal acquisition and visualization. All electrodes and components are custom-fabricated, eliminating the need for disposable parts while ensuring stable signal performance over extended use. Previous evaluations have confirmed the reliability of the electrodes, with performance comparable to standard clinical electrodes [22,23,24].

To validate the performance of the proposed wearable belt system, we conducted a comparison study to assess its signal quality relative to established clinical-grade acquisition systems. Healthy participants completed a standardized protocol that included tasks designed both to replicate secondary effects often associated with IBS, such as stress-induced autonomic changes and abdominal muscle activation, and to reflect typical movements encountered in daily life. These tasks included cognitive stress (color-word interference), postural adjustment (tilt table test), targeted muscular activation (abdominal contraction), and a combined multi-stressor condition. During each phase, biosignals were recorded simultaneously using both the custom-built wearable system and corresponding reference devices commonly used in research and clinical settings.

Each signal modality on the belt system was evaluated against its respective benchmark system. Quantitative indices were extracted from the recorded signals to assess waveform integrity, including metrics from both time and frequency domains. These indices were computed independently for both systems and compared to evaluate consistency in signal quality, shape, and responsiveness across different physiological states. In addition, we analyzed the robustness of each channel during tasks that naturally introduced substantial motion and postural changes, to evaluate the system’s practical reliability under real-world conditions. Statistical analyses were conducted where appropriate to compare the outputs across devices and verify the reliability of the wearable system.

### 2.1. Design and Implementation

#### 2.1.1. Physical Design of Belt and Wristband

**Figure 1:**
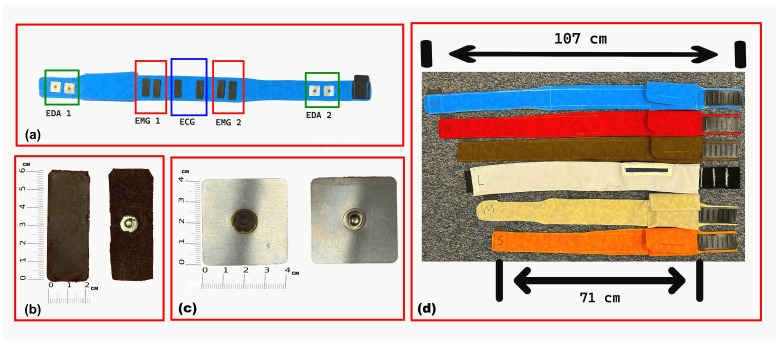
Overview of the developed wearable belt and electrode designs: (**a**) inside (skin-facing) view of the abdominal belt showing electrode locations for EDA, sEMG, and ECG; (**b**) custom-fabricated CB–PDMS electrodes with embedded copper mesh for sEMG and ECG acquisition; (**c**) CNC-fabricated stainless steel electrodes for abdominal EDA recording; and (**d**) available belt sizes from Small to XXXL in different colors.

The abdominal belt is fabricated from polyurethane (PU) synthetic fabric and features a cotton-woven backing that provides durability, water and fade resistance, and wrinkle resistance. Polyurethane was selected for its biocompatibility, flexibility, and comfort during prolonged skin contact. Six sizes were designed to accommodate user preferences and waist circumferences ranging from 71 cm to 107 cm. A proper fit promotes stable electrode–skin contact, which is essential for maintaining high-quality signal acquisition. The belt design includes multiple adjustable size options to accommodate children and adolescents, ensuring stable electrode contact across a wide range of body sizes. The belt’s skin-facing side, electrode types, and available sizes are illustrated in Figure 1.

**Figure 2:**
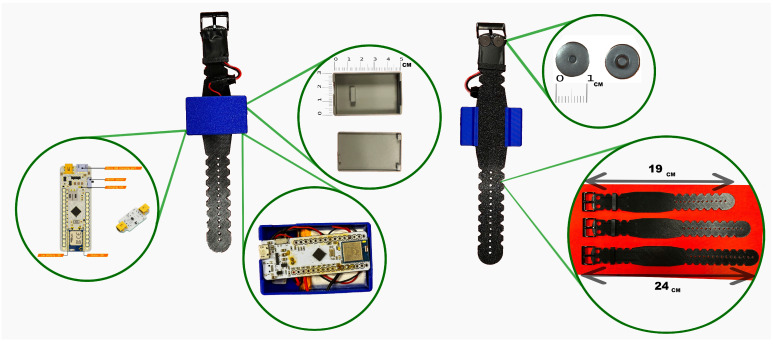
The designed wristband system with protective case housing the BITalino Mini acquisition board, showing three available wristband sizes, front and back views, and the Ag/AgCl electrodes used for wrist-based EDA acquisition.

All electronic components, including sensors, wiring, and circuit boards, are fully integrated into the belt, meaning that they are embedded within the belt’s structure rather than attached externally [25]. This design creates a compact and wireless system that minimizes user interference and allows comfortable, unobtrusive wear during a wide range of activities and body positions. The fabric surface of the belt can be wiped clean directly while the electronic components remain embedded, eliminating the need for disassembly. The wristband was designed as the primary site for EDA acquisition because the wrist reliably exhibits stronger and more consistent sympathetic responses than most other peripheral locations. However, because participants were already wearing the abdominal belt, we also integrated torso electrodes to serve as a complementary channel. This design creates a dual-site EDA system, where the wrist typically delivers higher-quality responses but can be vulnerable to motion artifacts, while the torso offers a more stable alternative during high-movement conditions. This configuration provides redundancy and reduces the likelihood of signal loss during arm movements, while adding minimal hardware complexity to the belt-based system. The complete wristband design, including case integration, available sizes, and electrode positioning, is shown in Figure 2.

The EDA electrodes are positioned on the underside of the wrist to target eccrine-rich regions for optimal signal quality. Its structure incorporates ABS components to enhance flexibility and minimize discomfort. The electronics, including the acquisition board, are integrated into a top-mounted case, resulting in a compact and fully wireless configuration that supports unobtrusive, continuous use in real-life scenarios.

#### 2.1.2. Hardware Configuration and Custom Electrode Fabrication

**Figure 3:**
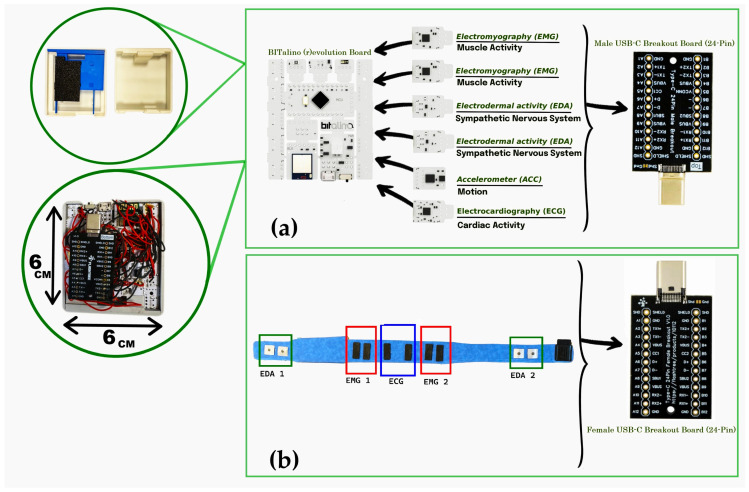
Internal electronics of the abdominal belt: (**a**) belt-side circuitry showing the BITalino (r)evolution Plugged Kit BLE/BT board with custom-fabricated biosignal sensors and integrated USB Type-C male breakout board for sensor interfacing; (**b**) belt-side circuitry with the USB Type-C female breakout board soldered via internal wiring to the embedded ECG, sEMG, and EDA electrodes.

The electronic architecture of the system was built upon the BITalino (r)evolution Plugged Kit BLE/BT, which served as the main acquisition board within the abdominal belt. This board contains 10 sockets, of which six are functional for biosignal acquisition. These were allocated to two EDA channels, two EMG channels, one ECG channel, and one ACC channel, enabling synchronized multimodal monitoring [26,27]. To achieve a compact layout suitable for wearable integration, the original female sockets on the board were removed. Sensor leads were then securely soldered directly to the board, ensuring robust electrical connections and minimizing spatial constraints. An identical customization approach was applied to the wristband, which used a smaller BITalino Mini board configured for a single EDA channel acquisition. The internal electronic layout of the belt, including the BITalino (r)evolution board, custom sensors, and USB Type-C interface, is shown in Figure 3.

Both the belt and wristband featured custom-designed protective cases to house the electronics. In the belt configuration, the case consisted of three stacked levels. The first level housed the BITalino main board; the second level contained the biosignal sensors (EDA, ECG, and EMG); and the third level included the power and interface components. Each layer was mechanically separated using laser-cut ABS plates for structural stability and electrical isolation.

For connectivity and modularity, the sensors and power system were interfaced using a USB Type-C connection. A USB Type-C Male Breakout Board (24-pin, gold-plated) was integrated into the third level of the belt’s case and connected to all sensors. Ground lines from all channels were interconnected at this stage. This male connector interfaced with a matching USB Type-C Female Breakout Board positioned externally within the belt structure. The female connector was soldered via internal wiring to the respective electrodes embedded in the belt. This modular setup enabled easy detachment of the main electronic module from the belt for maintenance or cleaning, using a standard USB-C cable for reconnection.

The ECG and sEMG electrodes were fabricated in-house as identical rectangular CB–PDMS pads (6 × 2 cm) with embedded copper mesh, following the fabrication method described in a previous study [23], to ensure flexibility, biocompatibility, and stable conductivity for integration into the wearable system. Their mechanical durability and electrical performance have been validated in previous studies, showing signal quality comparable to conventional gel electrodes [28,29]. For abdominal EDA recording, square stainless-steel electrodes (3.5 × 3.5 cm) were used. These electrodes were mounted securely onto the belt with internal wiring to maintain a compact, unobtrusive design. For wrist-based EDA, commercial snap-type Ag/AgCl electrodes were selected due to their high signal sensitivity and skin compatibility. Soldering connections to Ag/AgCl surfaces posed technical challenges, which were resolved using Kester 331 organic flux and 63/37 Sn/Pb rosin-core solder wire to ensure a stable and low-noise electrical connection without compromising electrode integrity.

#### 2.1.3. Electrode Placement and Anatomical Rationale

Electrodes were strategically positioned to optimize signal quality for monitoring visceral pain in IBS, guided by anatomical landmarks and physiological relevance to autonomic and neuromuscular responses [30].

For ECG acquisition, electrodes were placed on the lower abdomen, with positive and negative electrodes positioned bilaterally, 3 cm apart, over the rectus abdominis muscle on either side of the umbilicus. This arrangement facilitated a modified lead II configuration tailored to the belt’s form factor. A shared reference electrode, also used for abdominal EDA, was placed in the lumbar region to minimize interference from muscle activity and maintain signal grounding.

sEMG electrodes targeted neuromuscular activity linked to IBS-related visceral hypersensitivity and altered colonic motility [31]. Four electrodes, arranged in two bilateral pairs, were placed over the external oblique muscles in the lower abdominal quadrants, corresponding to the ascending and descending colon regions. This placement captured muscle contractions reflective of localized neuromotor responses, providing a noninvasive correlation to internal gastrointestinal dysfunction.

**Figure 4:**
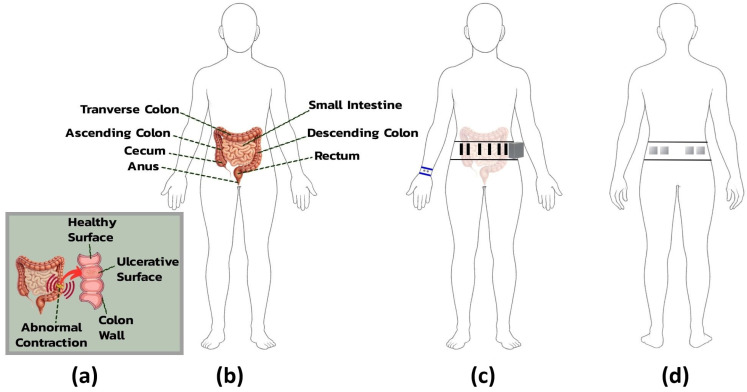
Anatomical views and sensor placement: (**a**) intestinal surface changes in IBS, healthy, and ulcerative conditions; (**b**) anterior view of small and large intestines with labeled segments; (**c**) body wearing the abdominal belt (ECG, sEMG) and wristband (EDA); and (**d**) posterior belt view showing EDA electrode placement.

EDA was primarily measured using wristband electrodes placed on the volar wrist, a site rich in eccrine sweat glands and well suited for detecting high-amplitude responses to emotional or cognitive stress. As the belt was already part of the system design, two additional abdominal electrodes were included as extra channels to provide backup measurements with stable baseline conductance and reduced gastrointestinal interference. Figure 4 provides an anatomical overview of IBS-related abdominal structures and the rationale for electrode placement on the belt and wristband.

### 2.2. Hardware Validation

#### 2.2.1. Battery Runtime Test

**Figure 5:**
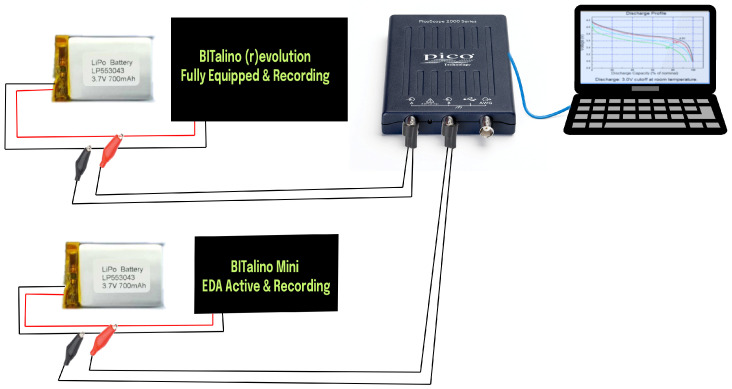
Diagram showing PicoScope 2000 setup for logging battery voltage of the wearable system under full load.

To monitor battery discharge behavior over an extended duration, we employed the PicoScope 2000 series, a compact USB oscilloscope capable of long-term data logging with adjustable sampling rates, selected for its ability to continuously record voltage data and export it in CSV format, enabling post-acquisition analysis of battery performance without data loss or overflow [32]. The voltage monitoring was conducted noninvasively by probing the battery terminal voltage while the system was operating under full load, with Channel A of the PicoScope connected across the terminals of the YDL 3.7V 700mAh 603035 rechargeable and replaceable lithium-polymer battery, using standard oscilloscope probes with the ground clip attached to the battery’s negative terminal and the signal tip connected to the positive terminal. The PicoScope was configured in DC-coupled mode to ensure accurate tracking of voltage drop. During the test, the wearable belt system was fully active with all biosignal channels enabled, including two EDA, one ECG, two EMG, and one ACC, alongside continuous Bluetooth streaming, and the system remained operational until automatic shutdown occurred due to voltage decay below the BITalino board’s cutoff threshold, typically around 3.3 V. The PicoScope 2000 setup for battery runtime monitoring is shown in Figure 5.

#### 2.2.2. Electrode Impedance Test

To characterize the electrical properties of the custom-fabricated electrodes and evaluate their suitability for biopotential acquisition, an impedance spectroscopy test was conducted using the Hioki IM3570 Impedance Analyzer across a frequency range of 20 Hz to 10 kHz. This method provides insight into the capacitive and resistive behavior of the electrode–skin interface, which is critical for ensuring signal fidelity, minimizing motion artifacts, and optimizing electrode material and geometry. The electrode impedance measurements were not intended for material comparison but to confirm that each electrode, as implemented in its final configuration and anatomical placement, operates within the optimal impedance range for its target signal. Prior comparative studies have established the relative performance of these materials; our goal here was to validate their suitability in the specific geometries and skin sites used in the belt and wristband designs. The selected frequency band encompasses the spectral range relevant for EDA, sEMG, and ECG, enabling a comprehensive evaluation of electrode performance for multimodal biosignal acquisition.

Measurements were performed in a two-electrode configuration under controlled skin preparation and contact pressure conditions to minimize variability. Each electrode was tested on hydrated skin to mimic real-world conditions encountered during wearable operation. The impedance analyzer applied a low-amplitude sinusoidal excitation signal, and both the real and imaginary components of impedance were recorded to compute magnitude and phase across the tested frequencies. The resulting impedance spectra were plotted to visualize frequency-dependent characteristics and to compare the performance of different electrode types, including CB–PDMS, stainless steel, and Ag/AgCl electrodes.

### 2.3. Experimental Protocol

The experimental protocol was developed to evaluate how well the wearable system could capture high-quality physiological signals during structured tasks that simulate real-life stressors common in individuals with IBS [33]. The study was approved by the Institutional Review Board at the University of Connecticut and was conducted to validate both the technical performance and clinical applicability of the multimodal wearable system. The protocol was designed to simulate autonomic and somatic responses relevant to IBS pathophysiology, ensuring clinical relevance without requiring symptomatic testing. Because IBS manifestations such as visceral hypersensitivity and autonomic dysregulation are not uniform across patients, relying on symptom-driven variability would confound the evaluation. Healthy participants therefore provide a reproducible and controlled framework for validation before applying the system to IBS research [34,35,36]. Twenty healthy adult participants gave informed consent before joining the study. To reduce variability from external factors, participants were instructed to avoid caffeine and alcohol for 24 h and to fast for at least three hours before the session.

All testing was conducted in a quiet, temperature-controlled laboratory. After arrival, participants rested in a supine position for three minutes to establish a baseline. The abdominal belt and wristband were then applied, and skin areas designated for electrode placement were cleaned with alcohol wipes to lower skin impedance. Electrode positions and system configuration were kept consistent across all subjects.

**Figure 6:**
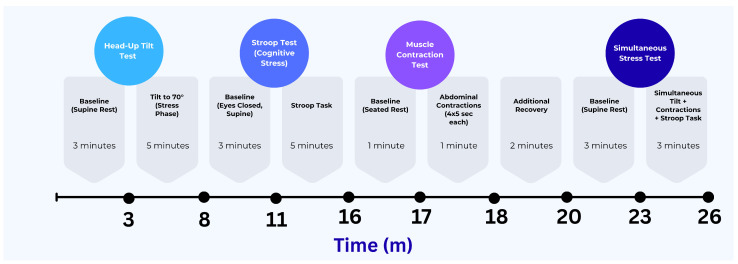
Timeline of experimental protocol with epochs for baseline and four task conditions.

The protocol included four tasks aimed at triggering distinct physiological responses: a passive head-up tilt to activate cardiovascular autonomic regulation, a Stroop test to introduce cognitive stress, an abdominal contraction task to engage local muscle activity, and a combined task that involved both cognitive and physical challenges under orthostatic stress. Each task began after a brief baseline recording period. These conditions were chosen to test the performance of all sensing modalities, and motion under both isolated and combined physiological activations. The experimental protocol timeline and analysis epochs are depicted in Figure 6.

Signals from the wearable system were recorded using the OpenSignals mobile app, while reference signals were simultaneously captured using laboratory-grade equipment. Manual event markers were used to synchronize the datasets during post-processing. The protocol was applied consistently for every participant to validate the system’s comfort, durability, and signal quality across various physiological conditions.

### 2.4. Signal Processing and Data Analysis

#### 2.4.1. EDA Analysis

Raw EDA signals were preprocessed to enhance signal quality and ensure accurate extraction of both tonic and phasic components. A low-pass finite impulse response (FIR) filter with a 1 Hz cutoff was applied to attenuate high-frequency noise, followed by a 1-s median filter to suppress sharp motion artifacts and transient discontinuities. After filtering, the signal was downsampled to 8 Hz, preserving relevant autonomic information while reducing data dimensionality for subsequent analyses [37]. MATLAB software was used for signal processing and metric analysis.

##### Time Domain Analysis

To extract sympathetic response features from the EDA signal, we used the non-negative sparse deconvolution algorithm known as SparsEDA [38]. This algorithm models the observed signal as a sum of a smooth tonic component and a sparse set of discrete phasic responses, using a physiologically inspired convolution model [38]. SparsEDA was applied to the entire continuous EDA recording before segmentation into protocol-defined intervals. For each 150-s segment defined within the experimental protocol, SparsEDA decomposed the EDA signal into its tonic background (skin conductance level, SCL) and a train of non-specific skin conductance responses (NS.SCRs). From these outputs, the average tonic level was computed over the window to represent the SCL, and the number of discrete phasic responses exceeding a threshold of 0.01 µS was used to quantify NS.SCRs, which reflect spontaneous sympathetic arousal events not directly tied to specific stimuli. This decomposition enabled clean isolation of autonomic response patterns associated with baseline and stimulus periods.

##### Frequency Domain Analysis

In addition to time-domain indices, two frequency-based features were computed to assess the sympathetic spectral content of the EDA signal. The first metric, EDASympn, represents the normalized power within the low-frequency band spanning 0.045 to 0.25 Hz. To obtain this, the filtered and downsampled EDA signal was processed using Welch’s power spectral density estimation with a 128-point Blackman window and 50% segment overlap. The resulting power spectrum was normalized, and the mean power within the specified frequency range was computed as the EDASympn index, which is known to correlate with sympathetic arousal during stress [3].

To capture time-varying sympathetic fluctuations, we also computed the TVSymp index using variable frequency complex demodulation (VFCDM) [39]. This technique decomposes the EDA signal into time-frequency components, isolating the 0.08–0.24 Hz band associated with dynamic sympathetic activity. The instantaneous amplitude of this band-limited component was extracted using the Hilbert transform and normalized to the standard deviation of the full EDA signal [4]. The average amplitude over the 4-min window was then used as the TVSymp value. This method provides a sensitive and temporally resolved measure of autonomic activation.

All preprocessing and feature extraction steps were identically applied to both abdominal (belt-based) and wrist (reference) EDA signals. This ensured consistency across locations and enabled reliable intra-subject comparison of signal quality and physiological responsiveness.

#### 2.4.2. EMG Analysis

Surface EMG signals were recorded using two bipolar channels to assess muscle activation patterns during abdominal contraction tasks. The signals were sampled at 1000 Hz and preprocessed to eliminate low-frequency artifacts and powerline interference. A high-pass filter with a cutoff frequency of 10 Hz was applied to remove motion-related baseline drift and slow physiological fluctuations. Additionally, a 60 Hz notch filter was implemented to attenuate line noise from ambient electrical sources. Both filters were implemented as 4th-order Butterworth IIR filters with zero-phase distortion via forward and reverse filtering. The filtered signals were retained for further time- and frequency-domain feature extraction [40].

##### Time Domain Analysis

The time-domain behavior of the EMG signal was analyzed to characterize the amplitude envelope associated with voluntary abdominal muscle contractions. Following preprocessing, the EMG signal was fully rectified by taking the absolute value of the waveform, transforming the zero-mean bipolar signal into a unidirectional time series representing signal magnitude. This rectified signal was then downsampled by a factor of 24, resulting in an effective sampling rate of 41.66 Hz, which preserves low-frequency amplitude modulations characteristic of human muscle activity. This downsampled signal served as the linear envelope and is commonly interpreted as a smooth proxy for the amplitude and duration of neuromuscular activation.

Linear envelope analysis was performed over task-specific epochs in which subjects were instructed to contract the abdominal muscles during defined test periods. The envelope was segmented according to these intervals, and the mean amplitude within each segment was computed to estimate muscle activation magnitude [41]. This approach enables consistent comparison of muscle effort across trials and subjects. The envelope provides a temporally smoothed representation of muscle activity and allows for visualization of activation patterns that align with protocol-defined tasks, such as single contractions or repeated strain efforts.

In addition to envelope analysis, the root mean square (RMS) of the EMG signal was also computed. RMS reflects the power of the EMG signal over a given time window and is widely used as a robust indicator of muscle activation intensity [42,43]. For each contraction segment, RMS values were calculated using 2 ms non-overlapping windows on the unrectified, filtered EMG signal. This metric is sensitive to both the number and firing rate of motor units and complements envelope amplitude in evaluating time-localized muscular activation levels.

##### Frequency Domain Analysis

To investigate the spectral characteristics of abdominal EMG signals, PSD analysis was conducted on the preprocessed signals using Welch’s method. Each PSD estimate was computed using 128-point Blackman windows with 50% overlap to balance frequency resolution and spectral variance [43]. The analysis targeted the 20–500 Hz band, which encompasses the dominant frequency components of surface EMG and excludes low-frequency motion artifacts and high-frequency instrumentation noise. PSD estimates were first obtained from unfiltered signals; however, additional analysis with band-pass filtering resulted in higher correlations, although the improvements remained modest.

The PSD plots provided insight into the energy distribution of EMG signals over frequency and were used to evaluate the presence of motion artifacts, noise contamination, and the spectral sharpness of the signal. To reduce DC bias and enhance the clarity of dominant peaks, the mean value of each signal was subtracted prior to PSD estimation. This preprocessing step ensured that most spectra exhibited a clearly defined dominant frequency within the 35–200 Hz range, facilitating more reliable spectral interpretation. Consequently, spectral energy distribution was used to qualitatively assess signal content, while quantitative noise-to-signal metrics were not emphasized in the statistical analysis.

#### 2.4.3. ECG Analysis

The raw ECG signal was first bandpass filtered using a 4th-order Butterworth filter with cutoff frequencies of 0.5 Hz and 50 Hz to remove baseline drift and high-frequency noise, including muscle and motion artifacts [44]. Peak detection was performed using the qrsdetect function from the BioSig MATLAB toolbox, which applies a non-linear energy operator-based QRS detection algorithm [45]. Each detected peak was visually reviewed to correct false positives or missed detections, ensuring an accurate R–R interval (RRi) series for subsequent HRV analysis. The instantaneous heart rate (HR) was computed by calculating the reciprocal of the time interval between successive R-peaks. This HR series was interpolated using cubic spline interpolation and resampled at 4 Hz to produce a continuous HR waveform suitable for both time-domain and frequency-domain analyses [46].

ECG signals were recorded continuously across all four protocol stages. HRV indices were computed for the Stroop and Head-Up Tilt (HUT) tests, where motion artifacts were minimal and clear R-wave morphology was preserved. For the Muscle Contraction and Simultaneous Stress tests, denoising was applied to remove the specific artifacts they exhibited, primarily motion and electromyographic (EMG) interference caused by voluntary and reflexive muscular contractions. To mitigate these artifacts, an adaptive denoising approach based on the Normalized Least Mean Squares (NLMS) algorithm was implemented. In this method, the filtered EMG channels (20–120 Hz) and the accelerometer (ACC) signal (0.1–5 Hz) were used as noise reference inputs. The adaptive filter iteratively estimated the correlated motion- and EMG-related components in the ECG and subtracted them from the primary signal in real time. To prevent morphological distortion of the QRS complexes, a QRS-gated adaptation scheme was employed, wherein the filter coefficients were frozen during R-peak intervals, ensuring that adaptation occurred only between beats. R-peaks were detected using a modified Pan–Tompkins algorithm applied to both the raw and denoised ECG signals. In the raw ECG, strong motion and muscle interference resulted in multiple false R-peak detections or missed beats. After applying the denoising procedure, the R-peaks were accurately localized, demonstrating the benefit of adaptive filtering under dynamic muscular conditions.

##### Time Domain Analysis

The time-domain HRV indices were derived from the RRi series computed during each task segment. The mean RR interval (meanRR) was used to estimate the average cardiac cycle length over each segment. The standard deviation of all normal-to-normal intervals (SDNN) and the root mean square of successive differences (RMSSD) were computed to assess overall HRV and short-term beat-to-beat fluctuations, respectively. Additional time-domain metrics included the number of adjacent RR intervals that differed by more than 50 milliseconds (RR50), and the percentage of RR intervals exceeding this threshold (pRR50). These indices collectively reflect both parasympathetic tone and the dynamic responsiveness of the cardiac system to physiological and psychological stressors [47].

##### Frequency Domain Analysis

Frequency-domain HRV features were calculated from the interpolated RR interval time series at 4 Hz using Welch’s periodogram method, with 32-point Hamming windows and 50% overlap [48]. The resulting PSD was integrated over the standard HRV frequency bands: low frequency (LF, 0.045–0.15 Hz) and high frequency (HF, 0.15–0.4 Hz). LF power reflects contributions from both sympathetic and parasympathetic activity, whereas HF power is primarily mediated by parasympathetic (vagal) influence. The LF/HF ratio was calculated as an index often interpreted as sympathovagal modulation. In addition, total power across the 0.003–0.5 Hz band was computed to quantify overall HRV during each task segment.

## 3. Results

### 3.1. Hardware Validation

#### 3.1.1. Battery Runtime Test Result

The belt system operated continuously for approximately 7 h, while the wristband sustained operation for approximately 10 h under full-load conditions. In both cases, real-time plotting in the OpenSignals mobile application was interrupted once the battery voltage declined to a level insufficient to maintain stable Bluetooth communication [49]. This voltage drop caused an increase in transient transmission errors, ultimately leading to the application terminating the live monitoring session.

**Figure 7:**
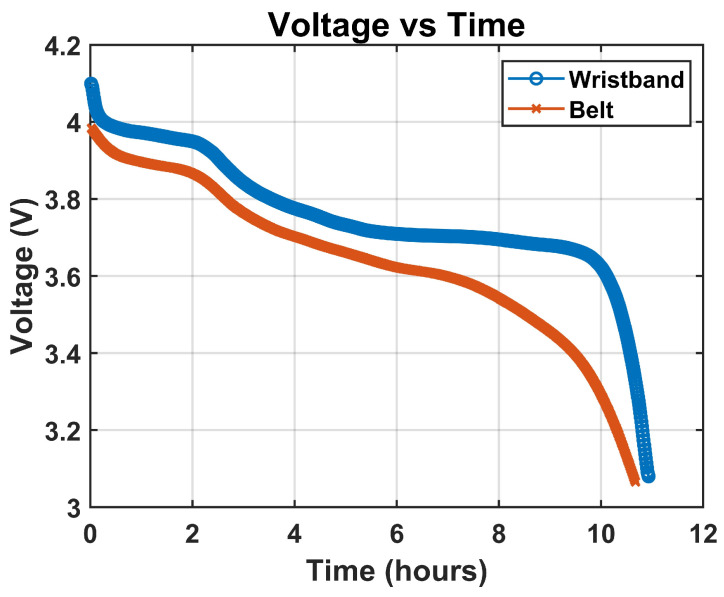
Battery voltage decay over time for belt and wristband devices under full-load operation.

Inspection of the voltage–time curves in Figure 7 showed a gradual decline throughout most of the discharge period, followed by an accelerated drop in the final phase prior to signal loss. The longer runtime observed in the wristband is consistent with its lower overall power consumption relative to the belt system, likely due to differences in channel configuration, processing load, and peripheral activity.

#### 3.1.2. Electrode Impedance Test Result

**Figure 8:**
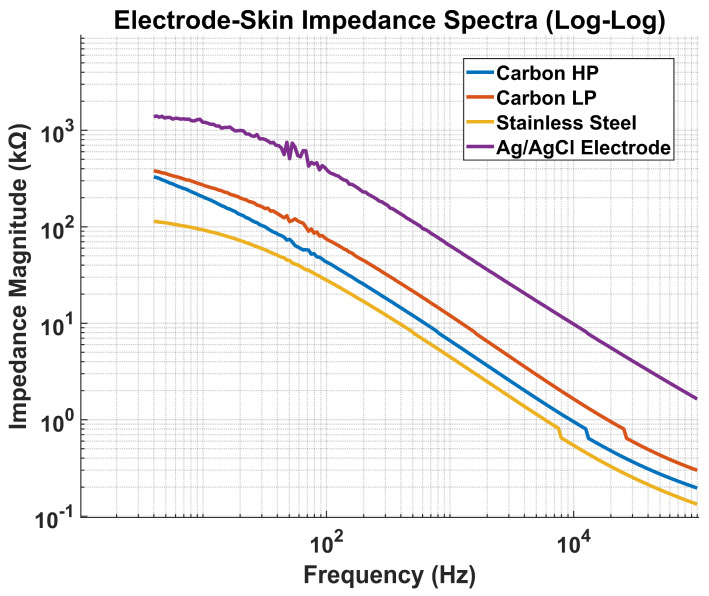
Electrode–skin impedance spectra for Ag/AgCl, CB/PDMS (high and low pressure), and stainless-steel electrodes.

Results for electrode–skin contact impedance measurements are presented in Figure 8. Measurements were obtained from the same subject, with Ag/AgCl electrodes positioned on the wrist, CB/PDMS electrodes positioned on the abdomen under both high and low contact pressure conditions, and stainless-steel electrodes positioned on the lumbus to replicate the spatial configuration of the belt and wristband systems. The frequency response was characterized over the range 4 Hz to 100 kHz.

The impedance profiles are consistent with trends reported in the previous literature [22,50], when accounting for electrode geometry and surface area. Stainless steel electrodes exhibited the lowest overall impedance; however, direct quantitative comparison across electrode types is limited by differences in anatomical placement and electrode dimensions.

### 3.2. Signal Quality Evaluation

#### 3.2.1. EDA Results

**Figure 9:**
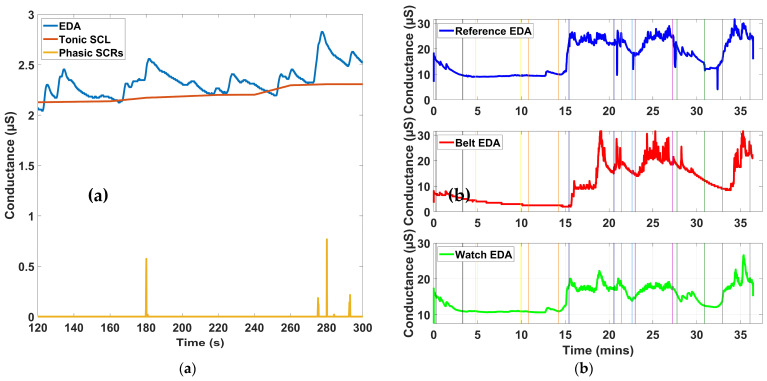
Comparison of EDA metrics between the prototype and reference device: (**a**) time-domain components including raw EDA (blue), SCL (red), and NS.SCRs (yellow); (**b**) Raw EDA data from both devices, lines indicate the four different tests.

Table 1 presents the EDA indices across baseline and test phases for the Tilt, Stroop, Contraction, and Combo conditions, while Figure 9 illustrates representative EDA recordings from both the prototype and reference devices.

For the time-domain indices, SCL in both the reference and prototype devices remained close during Tilt, with baseline and test values showing only small differences. In the Stroop condition, both devices showed an increase from baseline to test, with the prototype following the same trend but with slightly lower values than the reference during the active phase. In the Contraction condition, SCL rose from baseline to test in both devices, and the prototype values were consistently close to the reference, differing only slightly in magnitude. In the Combo condition, SCL increased in both devices from baseline to test, with nearly identical values at the test stage.

For NS.SCRs, the two devices tracked each other well across all conditions. In Tilt, baseline and test values were stable and closely matched. During Stroop, both devices showed higher values in the test stage compared to baseline, with the prototype capturing the same upward change as the reference. In Contraction, both devices recorded increases from baseline to test, with prototype values only slightly higher. In Combo, both devices showed low values at baseline and higher values in the test phase, again following the same trend.

For the frequency-domain index EDASympn, both devices produced nearly identical results across all four tests. Values remained stable between baseline and test for Tilt, Stroop, Contraction, and Combo, and the outputs from the prototype consistently overlapped with those of the reference. For the time-domain index TVSymp, both devices showed stable values during Tilt with minimal change from baseline to test. In Stroop, values increased from baseline to test for both devices, with close agreement between the two. In Contraction, both devices again demonstrated higher values during the active phase compared with baseline, with the prototype slightly above the reference but maintaining the same pattern. In Combo, both devices showed the largest increases from baseline to test, with prototype and reference values remaining close throughout.

#### 3.2.2. EMG Results

**Figure 10:**
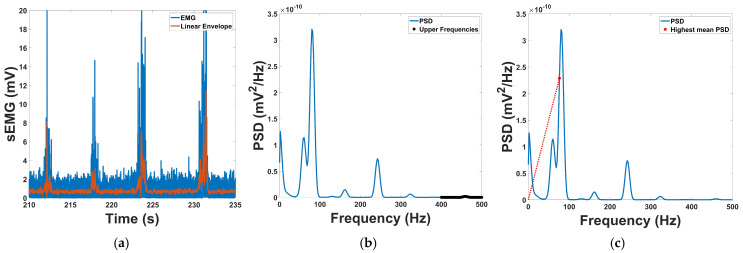
Illustrations of EMG signal quality assessment methods: (**a**) example of linear envelope reconstruction, with the red line showing the reconstructed envelope for a single unscaled EMG contraction; (**b**) SN ratio analysis, where black circles indicate high-noise frequencies used in SN ratio calculations; (**c**) SM ratio analysis, with the red dotted line marking the low-frequency cutoff, and power above this line identified as motion-related noise.

**Figure 11:**
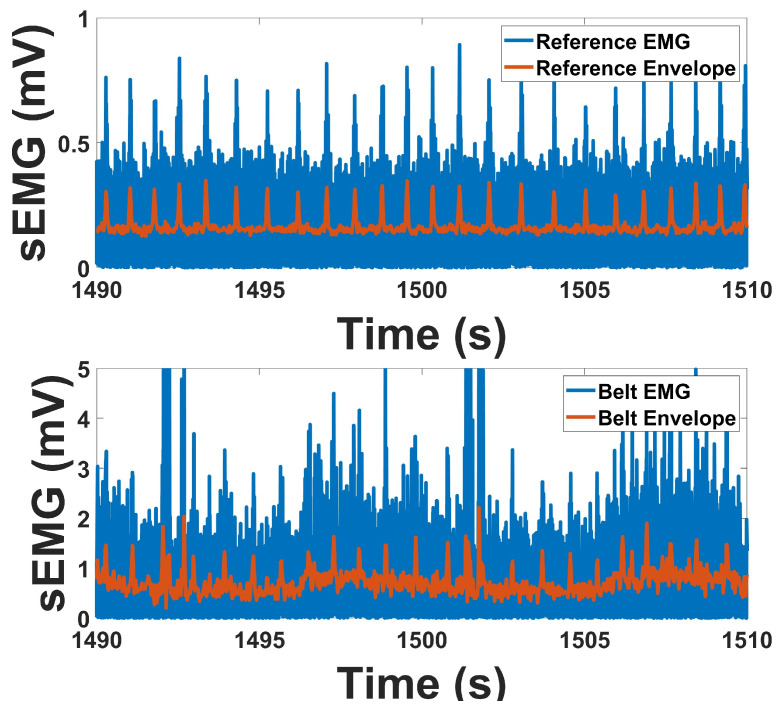
Side by Side comparison of EMG for both devices on one contraction, Envelope in Orange.

Table 2 summarizes the quantitative indices derived from both the reference and prototype devices during baseline and contraction conditions, while Figure 10 illustrates the EMG signal quality assessment methods. Figure 11 presents representative sEMG recordings, showing that both systems captured highly similar signal morphology during muscle contraction, with lower background activity at rest in the prototype device.

For the linear envelope amplitudes, both devices showed an expected increase from baseline to contraction. The prototype followed the same trend as the reference, with both left and right EMG channels rising substantially during contraction compared to baseline. Although the prototype reported higher absolute amplitudes, the progression from baseline to contraction was consistent across systems.

The envelope correlations averaged 0.79 across channels, confirming strong agreement in temporal signal shape between the prototype and reference. Similar correspondence was observed in RMS and PSD measures, supporting close alignment in both time and frequency domains.

The spectral indices further characterized the recordings. The reference device produced higher SN ratio, while the prototype showed increased signal-to-motion ratios (SM ratio), indicating better robustness against motion artifacts. Drop-in-power ratios were lower in the prototype but remained within a comparable range, reflecting preservation of the main spectral characteristics.

Overall, the EMG results demonstrate that the prototype reproduced the same activation patterns as the reference device, with strong correlations in envelope and spectral measures and consistent performance across contraction tasks.

#### 3.2.3. ECG Results

**Figure 12:**
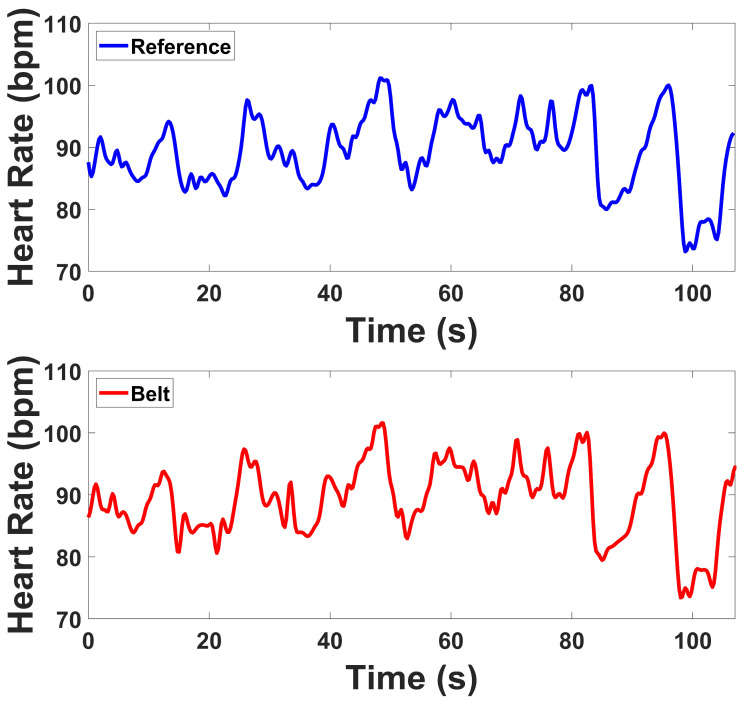
Side by Side comparison of HRV series for Prototype and Reference devices across baseline and test for Stroop Task.

**Figure 13:**
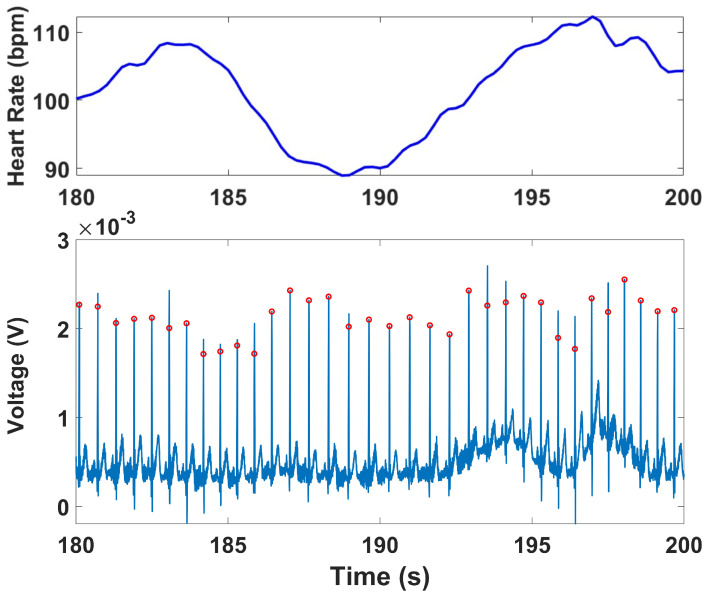
R-peak detection shown in red (**lower panel**) and results of the HR series calculated for ECG analysis (**upper panel**).

Table 3 presents the quantitative indices derived from the ECG recordings for the Tilt and Stroop tests. The Contraction and Simultaneous Stress tests involved substantial motion and EMG interference; therefore, HRV indices for these segments were computed after denoising and are reported separately in Table 4. Figure 12 shows representative HR calculations for both devices during the protocol, while Figure 13 illustrates representative ECG analysis results. For the time-domain indices, both reference and prototype devices demonstrated consistent decreases in mean RR intervals from baseline to test stages, reflecting the expected increase in HR during stress tasks. Measures of variability, including SDRR, RMSSD, RR50, and pRR50, followed the same trends across devices, with values from the prototype aligning closely with those of the reference. The prototype generally reported slightly higher variability values, but the changes between baseline and test were consistent with the reference device.

For the frequency-domain indices, both devices showed reductions in HF power from baseline to test during Tilt and Stroop, while LF values exhibited greater variation. Despite differences in absolute magnitude, both devices demonstrated similar sensitivity to changes between baseline and test stages. The LF/HF ratio increased from baseline to test in both Tilt and Stroop, with comparable patterns across devices. To further assess ECG performance under high-motion and muscular activity, additional analyses were conducted for the Muscle Contraction and Combination tests, which were previously excluded from HRV computation. During these tests, the ECG recordings exhibited substantial contamination from motion and electromyographic (EMG) interference due to strong abdominal and intercostal muscle activation.

**Figure 14:**
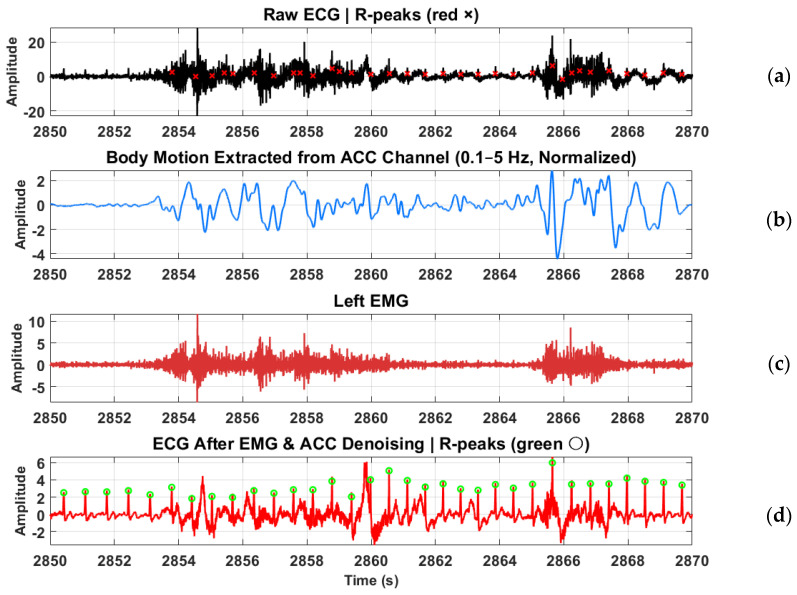
Example of ECG denoising during the Muscle Contraction test. (**a**) Raw ECG contaminated by EMG and motion artifacts; (**b**) accelerometer (ACC) signal showing body movement; (**c**) left EMG channel capturing muscular activity; (**d**) denoised ECG obtained using adaptive NLMS filtering with EMG and ACC as noise references. R-peaks were detected using a modified Pan–Tompkins algorithm, showing improved accuracy and stability after denoising.

R-peaks were detected using a modified Pan–Tompkins algorithm applied to both the raw and denoised ECG signals [51]. In the raw ECG, strong motion and muscle interference resulted in multiple false R-peak detections or missed beats. After applying the denoising procedure, the R-peaks were accurately localized, demonstrating the benefit of adaptive filtering under dynamic muscular conditions. Figure 14 illustrates this improvement.

Table 4 presents the HRV indices computed from the denoised ECG for the Contraction and Combination tests. Without denoising, these segments were unsuitable for HRV analysis due to severe signal degradation. After denoising, the ECG exhibited clear R-wave morphology, allowing the extraction of reliable RR intervals and computation of both time- and frequency-domain HRV indices. The recovered HRV parameters followed physiologically consistent trends across both tests, confirming that the denoising approach substantially improved ECG usability during high-motion conditions.

Across all experimental tasks, participants reported that the belt and wristband remained comfortable during use. No itching or skin irritation was reported by any subjects during or after device use, confirming its suitability for wearable biomedical applications.

## 4. Discussion

We developed and validated a novel integrated trimodal belt and wristband device capable of simultaneously recording EDA, sEMG, and ECG signals. The belt was specifically designed with electrode placement informed by the abdominal muscle anatomy most relevant to IBS, ensuring that signals are collected from physiologically meaningful sites. To complement torso acquisition, an EDA wristband was included, as the wrist has been shown to provide stronger EDA responses compared to abdominal sites, while the belt electrodes allow simultaneous multimodal capture from a single form factor [52]. In addition, the device was tested under battery operation, confirming that wireless use does not degrade signal quality. Together, these design considerations emphasize the system’s suitability for noninvasive monitoring of multimodal physiological activity in IBS populations.

In a series of cognitive, orthostatic, muscular, and combined stress tests, the wearable system demonstrated performance comparable to laboratory-grade reference devices across both time- and frequency-domain analyses. These findings confirm the feasibility of our device for real-world, continuous physiological monitoring.

For EDA, the wearable system (belt and wristband) closely matched the reference device across all four stress tests. Time-domain indices, including SCL and NS.SCRs, followed consistent baseline-to-task changes, with both devices showing similar increases during Stroop, Contraction, and Combo, while Tilt produced minimal shifts. Frequency-domain indices, including EDASympn and TVSymp, also showed parallel patterns across devices, with both detecting sympathetic activation during stress tasks. Overall, the prototype tracked the reference closely in both tonic and phasic responses, with only minor differences in magnitude between devices, supporting its validity for autonomic monitoring.

For sEMG, prototype and reference signals demonstrated strong agreement in both temporal and spectral measures. Envelope correlations averaged above 0.76, and RMS correlations were in a similar range, indicating that temporal activation patterns were well preserved. PSD correlations were also consistent, exceeding 0.72 across channels. Baseline-to-contraction amplitude changes were robust in both devices, with clear increases during contraction. Spectral indices revealed lower SN ratios in the prototype compared to the reference, while SM ratio were higher, and DP ratio were within a comparable range. These outcomes indicate that although the prototype exhibits modest differences in noise-related indices, its overall performance in capturing contraction-related EMG activity remains highly consistent with the reference. While the prototype demonstrated a higher signal-to-motion ratio compared to the reference, this outcome primarily reflects improved mechanical and electrical stability during abdominal motion rather than attenuation of the physiological muscle signal. As this study represents the first stage of validation for IBS-focused wearable monitoring, future iterations will incorporate enhanced electrode and amplifier designs to further optimize EMG sensitivity while preserving motion robustness.

For ECG, time-domain indices demonstrated strong agreement. Both devices showed reductions in MeanRR and related variability indices when transitioning from baseline to task, reflecting expected autonomic responses during Tilt and Stroop. SDRR and RMSSD values from the prototype were slightly elevated compared to the reference but maintained the same direction of change, while RR50 and pRR50 closely matched between systems. Frequency-domain indices revealed that LF and HF power were somewhat higher in the prototype, while Total Power also showed slightly inflated values. However, the LF/HF ratios were nearly identical between devices, and both tracked the same baseline-to-test transitions. These results confirm that the prototype is sensitive to autonomic changes, with generally consistent HRV metrics despite some magnitude differences in spectral power. Under high-motion and muscular activation, such as during contraction and combined stress conditions, the prototype continued to capture physiologically meaningful cardiac dynamics despite the presence of motion and EMG interference. The observed reductions in HRV indices reflected expected autonomic adjustments, indicating that the system remains sensitive to sympathetic activation and vagal withdrawal even in challenging conditions. This ability to preserve reliable cardiac variability measures under dynamic, real-world movements highlights the robustness of the device and supports its applicability for ambulatory and stress-related physiological monitoring. Although the lower-abdominal lead placement does not correspond to a standard limb or precordial lead, our comparison against the clinical reference device demonstrated consistent HRV features (MeanRR, RMSSD, LF/HF), confirming that this configuration yields physiologically valid autonomic indices.

Importantly, evaluation under the stress protocol demonstrated that the device maintained stable performance without degradation across signal modalities, even during battery-powered operation. Both time- and frequency-domain measures confirmed agreement with the reference devices, supporting readiness for untethered, real-world use. This robustness is critical for clinical translation, as ambulatory IBS monitoring requires reliable long-term performance.

Some discrepancies were observed that merit consideration. For EMG, the prototype exhibited a slightly lower signal-to-noise (SN) ratio than the reference but maintained strong motion robustness during contractions, likely due to the compact circuit layout and the proximity of EMG electrodes to the accelerometer, which supported real-time motion compensation. Future iterations could improve signal quality through optimized analog front-end filtering, enhanced shielding, and adaptive motion-denoising algorithms utilizing accelerometer data. In the ECG domain, the slightly higher variability in HRV metrics (e.g., SDRR, RMSSD, and pRR50) may result from electrode connections and motion sensitivity. Subsequent versions should refine the electrode–skin interface using flexible or textile-integrated dry electrodes and incorporate higher input impedance and baseline stabilization to improve robustness. For EDA, although strong correspondence was achieved with the reference system, further optimization of the AC-based excitation circuitry and electrode surface treatment could help maintain stable skin–electrode impedance during extended use.

The achieved 7–10 h runtime meets the needs of IBS-related laboratory protocols, which typically last only a few hours. With rechargeable and swappable batteries, the system can operate continuously throughout day-long sessions with minimal interruption. This represents a key proof-of-concept milestone for a fully integrated multimodal belt capable of reliable, wireless operation during extended experimental use. Future versions will focus on extending runtime for 24-h home and ambulatory monitoring.

Together, these findings support the wearable belt and wristband system as a robust platform for simultaneous, multimodal physiological monitoring. The ability to acquire EDA, ECG, and sEMG signals in parallel from anatomically relevant locations addresses a critical gap in IBS research, where no comparable wearable currently exists. Future studies should also examine whether multimodal feature integration improves diagnostic sensitivity and specificity relative to single-signal methods.

By integrating anatomically targeted, multimodal biosensing into a compact and reusable platform, this device addresses a significant unmet need in IBS research. Current objective, long-term physiological monitoring tools for IBS are limited, and our system offers a practical and comfortable solution. Its ability to concurrently capture autonomic and somatic responses provides a robust approach for objectively characterizing visceral pain and autonomic dysregulation in real-world settings.

Future work will focus on extending the system’s validation to diverse IBS patient populations, rigorously evaluating long-term comfort and durability, and integrating robust wireless data streaming capabilities for seamless clinical and home-based monitoring. The system provides objective, high-quality multimodal biomarkers that may serve as a foundation for future studies on visceral pain and autonomic dysfunction in IBS. The current findings establish the device’s technical validity and physiological reliability in healthy subjects, forming the groundwork for future investigations in clinical populations. By integrating anatomically targeted biosensing into a compact, wearable platform, this study introduces a proof-of-concept system designed to enable future development of objective, data-driven approaches for IBS assessment and management.

The primary goal of this work was to validate the accuracy and reliability of each sensing modality within the multimodal platform. The integration of fused-signal analysis and physiological-state classification represents a future stage of this research.

## 5. Conclusions

This study successfully demonstrated the development and rigorous validation of a novel wearable system, consisting of an abdominal belt and a wristband, designed for the simultaneous acquisition of EDA, sEMG, and ECG signals. Through a series of cognitive, orthostatic, muscular, and combined stress tasks, the system consistently exhibited signal quality and responsiveness comparable to established laboratory-grade reference devices. Critically, the belt maintained stable electrode-skin contact and provided reliable data acquisition under battery-powered operation, confirming its readiness for untethered, continuous use.

## Figures and Tables

**Table 1:** Results for EDA Indices ^1^.

		Tilt		Stroop		Contraction		Combo	
		Baseline	Test	Baseline	Test	Baseline	Test	Baseline	Test
SCL	Ref	9.153 ± 7.97	8.67 ± 8.64	10.03 ± 9.47	14.97 ± 9.45 *	14.65 ± 10.72	16.24 ± 9.61	15.10 ± 8.46	19.70 ± 11.37 *
	Pro	10.44 ± 5.28	9.87 ± 7.31	11.03 ± 10.45	12.60 ± 8.52 #	15.70 ± 11.22	17.78 ± 12.35	16.25 ± 11.87	19.63 ± 14.17
NS.SCRs	Ref	1.00 ± 0.61	1.05 ± 1.07	0.78 ± 0.79	2.16 ± 1.73 *	1.09 ± 0.86	1.92 ± 0.78	0.11 ± 0.22	3.22 ± 1.60 *
SCRs	Pro	0.89 ± 0.70	0.92 ± 1.14	0.78 ± 0.75	1.39 ± 1.36	1.52 ± 0.94	2.21 ± 0.96	0.83 ± 0.43 #	2.83 ± 2.01 *
EDA	Ref	0.43 ± 0.16	0.49 ± 0.01	0.49 ± 0.00	0.49 ± 0.01	0.50 ± 0.03	0.50 ± 0.02	0.49 ± 0.01	0.49 ± 0.01
Sympn	Pro	0.44 ± 0.16	0.50 ± 0.02	0.49 ± 0.01	0.49 ± 0.01	0.49 ± 0.01	0.49 ± 0.00	0.50 ± 0.04	0.49 ± 0.02
TV	Ref	0.25 ± 0.29	0.23 ± 0.23	0.24 ± 0.33	0.79 ± 0.72	0.44 ± 0.26	0.81 ± 0.46 *	0.32 ± 0.40	1.20 ± 0.67 *
Symp	Pro	0.28 ± 0.33	0.32 ± 0.30	0.27 ± 0.57	0.93 ± 1.02 *	0.53 ± 0.43	0.92 ± 0.60	0.41 ± 0.49	1.51 ± 1.10 #

^1^ Results for EDA-derived indices across different tests. Data are shown as mean ± SD. Symbols: * *p* < 0.05 versus baseline within the same device; # *p* < 0.05 versus reference device within the same stage. Abbreviations: SCL, skin conductance level; NS.SCRs, nonspecific skin conductance responses; EDASympn, normalized sympathetic EDA component; TVSymp, time-varying sympathetic index; Ref, reference device; Pro, prototype device.

**Table 2:** Results for EMG Indices ^1^.

Signals Interchangeability			Contraction Measure
	Amplitude Reference	BL	0.054 ± 0.012
		Test	0.443 ± 0.085 *
	Amplitude Prototype	BL Left EMG	0.161 ± 0.015
		Test Left EMG	1.523 ± 0.210 *
		BL Right EMG	0.158 ± 0.014
		Test Right EMG	1.487 ± 0.195 *
	Envelope Correlation	Left EMG	0.766 ± 0.210
		Right EMG	0.805 ± 0.207
	RMS Correlation	Left EMG	0.736 ± 0.164
		Right EMG	0.788 ± 0.172
	PSD Correlation	Left EMG	0.720 ± 0.190
		Right EMG	0.739 ± 0.111
sEMG indices			Contraction Measure
	SN ratio (dB)	Reference	19.843 ± 3.722
		Left EMG	11.687 ± 4.294 #
		Right EMG	14.742 ± 4.947 #
	SM ratio (dB)	Reference	3.7164 ± 4.391
		Left EMG	10.028 ± 5.244 #
		Right EMG	7.426 ± 4.070 #
	DP ratio (dB)	Reference	31.634 ± 1.503
		Left EMG	22.390 ± 5.722
		Right EMG	27.641 ± 5.382

^1^ Values shown as mean ± SD. * *p* < 0.05 vs. baseline; # *p* < 0.05 vs. reference device at same stage.

**Table 3:** ECG HRV Indices During Tilt and Cognitive (Stroop) Tests ^1^.

			Tilt		Stroop	
			Baseline	Test	Baseline	Test
Time Domain	MeanRR	Ref	0.87 ± 0.19	0.69 ± 0.11 *	0.9069 ± 0.2192	0.7906 ± 0.1613 *
		Pro	0.7770 ± 0.0948	0.64 ± 0.05 *	0.7517 ± 0.1175	0.7489 ± 0.1104 *
	SDRR	Ref	0.0867 ± 0.0750	0.05 ± 0.05	0.0919 ± 0.0620	0.0580 ± 0.0418
		Pro	0.102 ± 0.081	0.061 ± 0.056	0.110 ± 0.078	0.073 ± 0.050
	RMSSD	Ref	0.09 ± 0.091	0.04 ± 0.05 *	0.09 ± 0.07	0.04 ± 0.04
		Pro	0.120 ± 0.095	0.058 ± 0.060	0.118 ± 0.085	0.102 ± 0.048 *
	RR50	Ref	37.75 ± 37.68	11.1 ± 18.01	36.62 ± 27.02	23.25 ± 23.38
		Pro	58.50± 45.14	31.75 ± 51.03	62.12 ± 62.58	42.12 ± 34.23
	pRR50	Ref	29.02 ± 29.08	7.36 ± 12.21 *	29.57 ± 23.13	15.89 ± 15.81 *
		Pro	38.96 ± 30.32	17.07 ± 28.06	37.99 ± 33.39	26.52 ± 21.52
Frequency Domain	LF	Ref	20.73 ± 43.70	15.94 ± 18.27	20.163 ± 34.97	14.19 ± 25.16
		Pro	34.8 ± 52.0	27.2 ± 24.9	32.5 ± 40.2	25.4 ± 28.7
	HF	Ref	48.72 ± 102.19	23.61 ± 41.34	67.20 ± 143.58	32.673± 68.43
		Pro	72.5 ± 115.3	36.4 ± 52.9 #	91.3 ± 158.2	45.1 ± 75.6 #
	Total	Ref	4978.8 ± 10,766.3	2609.5 ± 3854.0	6569.48 ± 13,825.5	2887.17 ± 5455.0
		Pro	6120 ± 11,800	3420 ± 4420	7810 ± 14,200	3970 ± 5890 #
	LF/HF	Ref	0.57 ± 0.27	2.35 ± 1.43	0.81 ± 0.51	1.21 ± 0.92
		Pro	0.47 ± 0.41	1.745 ± 1.77	0.72 ± 0.45	0.81 ± 0.83

^1^ Values are reported as mean ± standard deviation. Symbols: * denotes a significant difference from baseline (*p* < 0.05); # denotes a significant difference from the reference device within the same stage (*p* < 0.05). Abbreviations: MeanRR, mean RR interval; SDRR, standard deviation of RR intervals; RMSSD, root mean square of successive differences between adjacent RR intervals; RR50, count of adjacent RR intervals differing by more than 50 ms; pRR50, percentage of RR50 relative to total RR intervals; LF, low-frequency power (0.045–0.15 Hz); HF, high-frequency power (0.15–0.40 Hz); Total, combined LF and HF power (0.045–0.40 Hz); LF/HF, ratio of LF to HF power; BL, baseline period; Test, stress test period; HUT, head-up tilt test; Ref, reference device; Dev, prototype device.

**Table 4:** ECG HRV Indices During Physical (Contraction) and Combined Stress Tests ^1^.

			Contraction		Combo	
			Baseline	Test	Baseline	Test
Time Domain	MeanRR	Ref	0.832 ± 0.130	0.712 ± 0.082 *	0.821 ± 0.141	0.584 ± 0.091 *
		Pro	0.817 ± 0.112	0.725 ± 0.074 *	0.818 ± 0.123	0.590 ± 0.086 *
	SDRR	Ref	0.082 ± 0.065	0.047 ± 0.038 *	0.079 ± 0.058	0.041 ± 0.036 *
		Pro	0.089 ± 0.070	0.052 ± 0.040 *	0.085 ± 0.061	0.046 ± 0.037 *
	RMSSD	Ref	0.071 ± 0.060	0.035 ± 0.029 *	0.068 ± 0.054	0.032 ± 0.027 *
		Pro	0.078 ± 0.064	0.039 ± 0.032 *	0.072 ± 0.057	0.035 ± 0.029 *
	RR50	Ref	26.50 ± 24.40	8.400 ± 10.350	22.60 ± 21.10	6.900 ± 9.850
		Pro	31.20 ± 26.37	10.8 ± 12.5	27.511 ± 23.90	9.300 ± 10.900
	pRR50	Ref	19.85 ± 18.40	5.620 ± 7.410 *	17.74 ± 16.90	4.910 ± 6.880 *
		Pro	23.40 ± 19.70	6.81 ± 8.20 *	20.90 ± 18.20	5.873 ± 7.300 *
Frequency Domain	LF	Ref	14.730 ± 23.60	11.210 ± 14.830	13.25 ± 21.74	10.150 ± 12.650
		Pro	17.954 ± 25.30	12.700 ± 15.617	15.0 ± 22.808	11.20 ± 13.3
	HF	Ref	33.149 ± 71.225	16.45 ± 32.12	29.320 ± 60.880	14.850 ± 28.947
		Pro	39.60 ± 74.50	18.96 ± 33.7	34.80 ± 63.90	16.2 ± 29.9 #
	Total	Ref	4380.20 ± 9656.30	2205.7 ± 3760.5	4178.9 ± 9335.8	2101.3 ± 3480.1
		Pro	4710.0 ± 9910.0	2320.4 ± 3890.4	4510.3 ± 9456.0	2310.76 ± 3590.0 #
	LF/HF	Ref	0.620 ± 0.380	2.740 ± 1.956	0.660 ± 0.440	3.053 ± 2.15
		Pro	0.690 ± 0.423	2.98 ± 2.10	0.740 ± 0.460	3.223 ± 2.280

^1^ Values are reported as mean ± standard deviation. Symbols: * denotes a significant difference from baseline (*p* < 0.05); # denotes a significant difference from the reference device within the same stage (*p* < 0.05). Abbreviations: MeanRR, mean RR interval; SDRR, standard deviation of RR intervals; RMSSD, root mean square of successive differences between adjacent RR intervals; RR50, count of adjacent RR intervals differing by more than 50 ms; pRR50, percentage of RR50 relative to total RR intervals; LF, low-frequency power (0.045–0.15 Hz); HF, high-frequency power (0.15–0.40 Hz); Total, combined LF and HF power (0.045–0.40 Hz); LF/HF, ratio of LF to HF power; BL, baseline period; Test, stress test period; HUT, head-up tilt test; Ref, reference device; Dev, prototype device.

## Data Availability

The raw data presented in this study are available on request from the corresponding author.

## Abbreviations

The following abbreviations are used in this manuscript:

ACC	Accelerometer
BL	Baseline
CB–PDMS	Carbon Black–Polydimethylsiloxane
DP ratio	Drop-in-Power ratio
ECG	Electrocardiogram
EDA	Electrodermal Activity
EDASymn	Normalized Sympathetic EDA Component
EMG	Electromyogram
HF	High-Frequency power
HR	Heart Rate
HRV	Heart Rate Variability
HUT	Head-Up Tilt Test
LF	Low-Frequency power (0.045–0.15 Hz)
LF/HF	Ratio of Low- to High-Frequency power
NS.SCRs	Non-Specific Skin Conductance Responses
PSD	Power Spectral Density
pRR50	Percentage of RR50 relative to total RR intervals
Ref	Reference Device
RMSSD	Root Mean Square of Successive Differences
RR50	Count of adjacent RR intervals differing by more than 50 ms
RRi	RR Intervals
SCL	Skin Conductance Level
SDNN/SDRR	Standard Deviation of RR Intervals
SM ratio	Signal-to-Motion ratio
SN ratio	Signal-to-Noise ratio
sEMG	Surface Electromyography
Test	Stress Test Period
TVSymp	Time-Varying Sympathetic Index
